# Effect of Lanthanum Oxide Addition on Microstructure and Wear Performance of Iron-Chromium Alloy Manufactured by Laser Direct Deposition Additive Manufacturing

**DOI:** 10.3390/ma15093234

**Published:** 2022-04-29

**Authors:** Yanhua Zhao, Wei Meng, Peifu Wang, Chuanbin Du, Xiaowei Wang

**Affiliations:** 1 School of Mechanical and Electronic Engineering, Shandong Jianzhu University, Jinan 250101, China; 2020075115@stu.sdjzu.edu.cn (W.M.); 2020070101@stu.sdjzu.edu.cn (C.D.); wxw221@163.com (X.W.); 2 Laser Institute, Shandong Academy of Sciences, Jinan 250103, China; 3 Jinan Special Equipment Inspection Institute, Jinan 250100, China; wangpeifu2001@sohu.com

**Keywords:** laser additive manufacturing, iron-chromium alloy, rare earth oxide, microstructure, wear performance

## Abstract

Additive Manufacturing (AM) has become increasingly common, and its use in various industries is increasing. However, the microstructure, friction and wear performance of metals made by AM, such as the inexpensive and relatively good-performing iron-chromium alloys, require further investigation. Generally, adding rare earth elements can effectively improve the performance of AM alloys, such as tensile strength, wear resistance, corrosion resistance, creep resistance, etc. This work aims to study the variation of microstructure, friction and wear properties of laser additive manufacturing processed iron-chromium alloys after adding different mass fractions of La_2_O_3_. The observations obtained by scanning electron microscopy showed that, with the addition of La_2_O_3_, the microstructure of AM alloy becomes more uniform and the grains are significantly refined. It is found by friction test that the running-in period is significantly shortened after the addition of La_2_O_3_. The coefficient of friction is reduced to a minimum of 0.68. Compared with AM alloys without La_2_O_3_, the wear rate of AM alloys with La_2_O_3_ is significantly reduced, with a maximum reduction of 38%. Using an optical microscope to observe the surface morphology of the wear scar, it is found that, after adding rare earth oxide, the wear mechanisms changed from adhesive wear and abrasive wear to abrasive wear, with the spalling of hard particles at the same time.

## 1. Introduction

Additive manufacturing (also known as 3D printing) is a revolutionary cutting-edge manufacturing technology that combines computer-aided design, material processing and molding technologies [1]. It uses the method of gradual accumulation of materials to manufacture solid parts [2,3]. Additive manufacturing can produce parts with complex structures in relatively short manufacturing cycles [4]. Therefore, additive manufacturing technology is the leading technology for the development of mass manufacturing mode to personalized manufacturing mode. More importantly, compared with traditional manufacturing processes, laser additive manufacturing of metal materials easily controls the composition of additive metal powders, which can effectively improve the performance of formed parts.

### 1.1. AM Alloy Performance Improvement

Many scholars have carried out laser additive experimental research on adding modifying elements to titanium alloys, aluminum alloys and other metal materials [5,6]. Yang [7] improved the grain characteristics of Ti6Al4V alloy by laser direct deposition technology by adding V element, and the tensile strength was improved. Qin [8] used selective laser melting (SLM) technology to prepare 7050 aluminum alloy with Zn content as high as 14%, and reported that the presence of Zn element increases the hardness and tensile strength of the samples. Sun [9] used laser direct deposition technology to prepare Inconel 625 alloys with different WC-12Co mass fractions. The results showed that increasing WC-12Co content can significantly improve the wear resistance of particle-strengthened samples. Hong et al. [10] and Shen et al. [11] added TiC element to Inconel625 alloy and studied the micro-manufacturing evolution mechanism and corrosion resistance of Inconel625 fabricated from laser additive manufacturing. Ge et al. [12] pointed out that the Al content has a significant impact on the hardness and yield strength of Ti6Al4V made by laser direct deposition AM. When the mass fraction of Al is 1.5%, the mechanical properties of AM alloys are comparable to those of Ti6Al4V itself. The performance is significantly improved. Cooper et al. [13] studied the effect of ceramic reinforcement relative to laser direct deposition to fabricate Inconel625 alloy. It is concluded that the addition of silicon carbide increases the hardness by 130% but also increases the porosity and crack rate of AM alloy. Zhao et al. also prepared a nickel-based tungsten carbide gradient coating by adding WC to the nickel-based alloy powder using the laser cladding additive technology, which effectively enhanced the hardness and wear resistance while reducing carking sensitivity [14].

### 1.2. Effect of Rare Earth Oxide on AM Alloy Properties

In addition to the above-mentioned additive elements, rare earths and their oxides are also often used as heterogeneous additive elements to improve the properties of additively manufactured alloys. This is because the fluidity of the molten pool is improved after adding rare earth elements, and the microstructure of the alloy is optimized, so pores and microcracks in the structure can be effectively reduced [15]. Wang et al. [16] studied the influence of adding CeO_2_ on the microstructure and main properties of laser cladding Ni60 on aluminum alloy surface. The results showed that CeO_2_ can optimize the microstructure of the Ni60 alloy fabricated from laser cladding, improve the uniformity of the grains of the alloy layer and effectively improve the corrosion resistance of the Ni60 alloy. Gu et al. [17] and Zhang et al. [18] prepared high-strength aluminum alloys by adding rare earth elements (Sc, Zr) using SLM technology, and the tensile strength after aging treatment can reach 531.1 MPa. The study by Yang et al. [19] showed that adding rare earth (Ce) can increase the mechanical properties and creep resistance of AM Zn implants. Banoth et al. [20] reported the effect of yttrium on the high temperature cracking and creep behavior of Hastelloy-X alloys fabricated from SLM. The experimental results showed that the cracks became more pronounced after adding yttrium, but the creep life is prolonged.

Lanthanum oxide, one of the rare earth oxides, is an inorganic compound, usually white powder. It is often used as an added element for material modification and has a wide range of uses. For example, lanthanum oxide can be used in the manufacture of piezoelectric materials, optical glass, etc. It can be used in electroforming electrode materials, which can effectively improve the resistance to galvanic corrosion. Moreover, lanthanum oxide is also often used as a heterogeneous additive element to improve the performance of AM alloys by analyzing existing research. Wang et al. [21] analyzed the influence of adding La_2_O_3_ particles on the microstructure and tensile strength of wire-fed AM TC4 alloys and reported that, as the mass fraction of La_2_O_3_ increases, the tensile strength monotonically increases, while the elongation decreases monotonically. Wang et al. [22] fabricated high-strength commercial pure titanium by adding La_2_O_3_ based on SLM technology. The improvement of mechanical properties such as yield strength was attributed to the synergistic effect of grain refinement strengthening and dislocation strengthening. Chen et al. [4] added lanthanum oxide particles of different sizes when using wire arc AM Al-Si alloys. The test results showed that the addition of nano-scale lanthanum oxide can effectively improve the grain structure of the alloy, and the tensile strength is significantly enhanced.

The influence of adding La_2_O_3_ on the strength and wear behavior of Nickle-titanium alloy made by laser direct deposition was investigated by Lu et al. [23]. The results showed that La_2_O_3_ can play a role in improving the strength. That is, after adding lanthanum oxide, the grains of the nickel-titanium alloy were refined and the wear resistance was improved. Shi et al. [24] investigated the effect of lanthanum oxide content on the wear resistance of laser cladding Ni60a/SiC coatings. It is showed that the friction coefficient and wear depth of coatings with 2 wt% La_2_O_3_ addition were 0.63 and 15.79 μm, respectively, which were 3% and 86% lower than those of the 65Mn matrix, respectively. Compared with the other samples, the coating demonstrated the best wear resistance. Zhao et al. [25] prepared laser cladding nickel-based coatings by adding lanthanum oxide. The test results showed that the wear resistance of the coating was increased by 4 times compared with the base 30CrMnSiNi2A steel. Zhang et al. [26] fabricated a nickel-based alloy coating with a lanthanum oxide content of 1.5% on a 1045 steel substrate using thermal spraying and conducted friction performance analysis. It was noted that the wear rate of the coating slightly decreased with sliding speed.

In summary, adding heterogeneous elements can effectively enhance the microstructure and mechanical properties of additive manufacturing metals. Lanthanum oxide is one of the most commonly used modifier elements for AM alloys. Most of the existing research using lanthanum oxide as an additive element to improve the properties of alloys focus on strength, corrosion resistance, etc., and most of the alloys targeted are titanium alloys, nickel-based alloys and aluminum alloys. Research on improving the wear resistance of AM iron-based alloy is relatively rare.

### 1.3. Scope of This Research

The mining industry has an urgent need for efficient and safe production of fully mechanized mining equipment. Effectively improving the wear resistance of typical parts such as hydraulic supports, sprockets and middle plates of scraper conveyors is of great significance to ensure their service reliability. Therefore, it is urgent to develop an iron-based alloy powder with high wear resistance and low cost for laser additive manufacturing, repair or surface strengthening of key parts of mining equipment. An iron-chromium alloy powder with high corrosion resistance which is inexpensive and can be used in laser additive manufacturing was investigated in our group’s previous work [27]. In this work, it is planned to further improve its wear resistance on the basis of this iron-chromium alloy. This paper aims to improve the grain structure and wear resistance of iron-chromium alloy by adding lanthanum oxide.

Therefore, iron-chromium alloys with different mass fractions of lanthanum oxide were fabricated by laser direct deposition. The effect of lanthanum oxide on the microstructure and element distribution was analyzed. The influence of lanthanum oxide content on the friction coefficient and wear rate of AM alloys was analyzed experimentally, and the wear mechanism was analyzed by the wear surface characteristics.

## 2. Experimental Details

### 2.1. Materials

**Figure 1:**
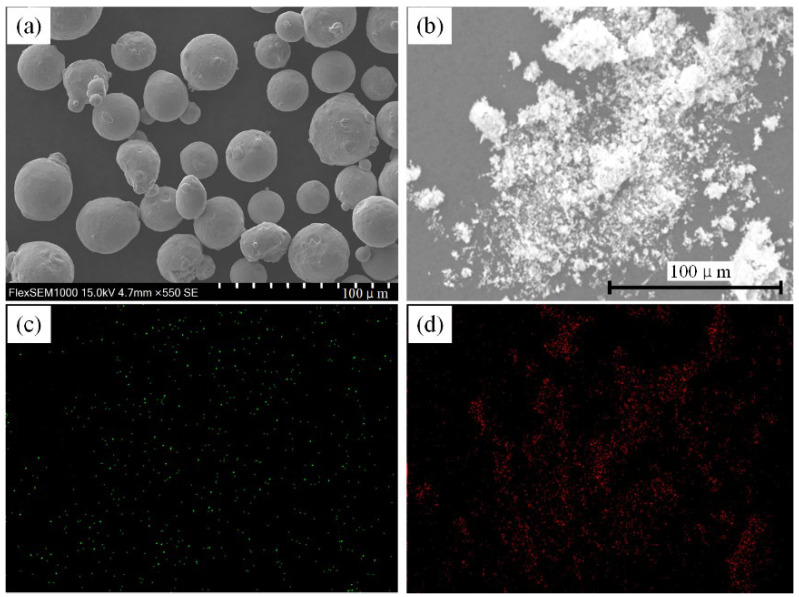
Microstructure of powders. (**a**) iron-chromium alloy powders, (**b**) La_2_O_3_ powders, (**c**) distribution of La element in La_2_O_3_ powders, (**d**) distribution of O element La_2_O_3_ powders.

The chemical composition of iron-chromium alloy powder is indicated in Table 1. The dimensions of the powder are 15 to 38 μm. The size of La_2_O_3_ particles is approximately 1 μm–5 μm. Different mass fractions of La_2_O_3_ were added, respectively, and iron-chromium alloy powders with La_2_O_3_ were produced after 72 h ball milling and mixing. The mass fractions of La_2_O_3_ content in the iron-chromium alloy powder were without La_2_O_3_ (Specimen-0), 0.5 wt% (Specimen-1), 1.0 wt% (Specimen-2), 1.5 wt% (Specimen-3) and 2.0 wt% (Specimen-4). The microstructure of the iron-chromium alloy powder particles and La_2_O_3_ powders is exhibited in Figure 1.

### 2.2. Experimental Processes

The iron-chromium alloys with rare earth oxide were fabricated by laser direct deposition additive manufacturing at the optimized process parameters, which were reported in our previous work [27]: laser power of 1.5 kW, scanning speed of 500 mm/min, lap rate of 30%, layer thickness of 0.4 mm. In the previous research, the iron-chromium alloy obtained by AM using these parameters has no defects such as pores and cracks and has high corrosion resistance, which can be applied to the repair and remanufacturing of compressor blades. The wire-cut and ultrasonically cleaned AM alloys were prepared into specimens using hot mounting. Then, the specimen was sanded with 320#, 1200#, 2000# sandpaper in turn until the surface was smooth and free of rough scratches. The surface of the specimen was mechanically polished with abrasive paste until the surface reached a mirror finish. In order to show the metallographic structure more clearly, the ground and polished alloy samples were chemically etched using a mixed reagent of HCl, HNO_3_ and H_2_O in a volume ratio of 1:1:1. A Scanning Electron Microscope (SEM, FEI Quanta FEG 250, Hillsboro, OR, USA) was used to observe the microstructure of AM alloys. Using the method of random sampling in a fixed area to calculate the average value, Image-Pro-Plus6.0 software (Media Cybernetics, Inc., MD, USA) was used to measure the average grain size of each specimen, and the average grain size and error value were calculated. Chemical element distribution was tested using the Energy Dispersive Spectrometer (EDS, FEI Quanta FEG 250, Hillsboro, OR, USA).

**Figure 2:**
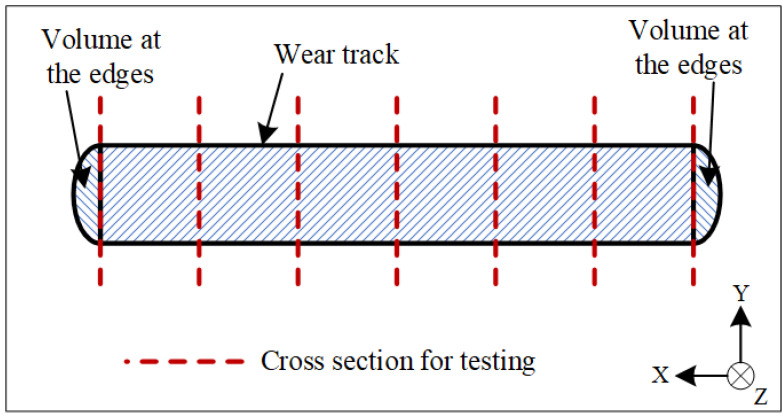
Schematic diagram of the wear scar used to calculate volume loss.

The dry sliding friction test was carried out on AM alloy samples using the friction and wear testing machine (UMT-2) at room temperature and without lubrication. A silicon nitride ceramic ball with a diameter of 9.525 mm was used as the counter-grinding pair. During the test, a 15 N vertical load was applied to the surface of the specimens; the sliding speed was 5 mm/s and the total time was 15 min. And coefficient of friction curve was automatically recorded. The microhardness of each specimen was tested using a MH-6 Vickers digital microhardness tester with a load of 10 g and a dwelling time of 5 s. The averaged results of three repeat tests were used in this article. The wear scar profile was observed using a white light interferometer (Veeco NT9300, Plainview, NY, USA). The scanning resolution of the white light interferometer is 0.01 μm in the Z direction and 0.01 mm in the X and Y directions. The wear volume loss was then estimated using MATLAB using the measured wear scar profile data. The primary wear track volume was estimated as the average cross-sectional area of seven profiles showed by the dash line in Figure 2. The worn surface morphologies were analyzed using an optical microscope (OM).

## 3. Results and Discussion

### 3.1. Microstructures

**Figure 3:**
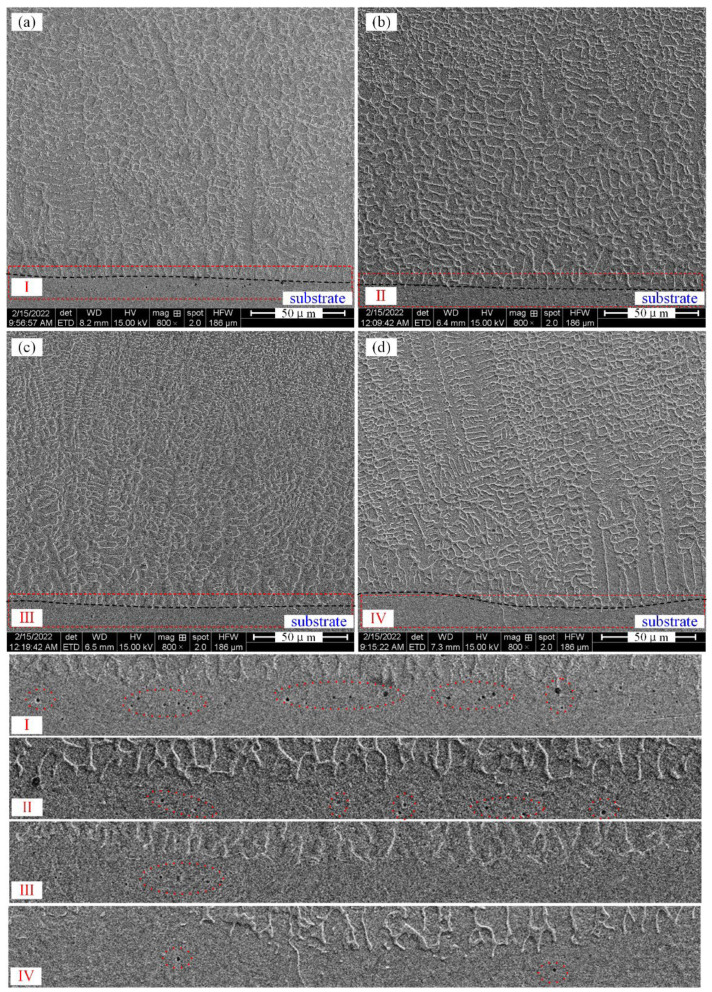
Microstructure near fusion lines of AM alloy deposits. (**a**) Specimen-1, (**b**) Specimen-2, (**c**) Specimen-3, (**d**) Specimen-4.

The microstructure of AM alloy specimens near the fusion line is shown in Figure 3. It can be clearly observed that AM alloy is free of pores and cracks. At the same time, the growth of AM alloy can be clearly specified and observed, with each sample having similar crystal growth characteristics. AM alloys grow upward from the fusion line. During the solidification process of the AM alloy, with the increase of the solidification rate and the decrease of the temperature gradient, cellular growth, cellular dendrite growth and equiaxed dendrite growth can be found in sequence.

More importantly, the existence of pores can be observed below the fusion line and on the side of the substrate, as shown in the red dash line in Figure 3I–IV. The reason for the formation of pores is that the residual gas is heated and expanded inside the alloy and continuously gathers to form bubbles floating up. Before it solidifies rapidly, it cannot escape in time and is sealed in the interface near the fusion line. It can be seen that the number of pores is larger when the mass fraction of La_2_O_3_ is 0.5 wt% (Specimen-1), and the number of pores decreases gradually as the mass fraction of La_2_O_3_ increases. This shows that the addition of rare earth oxide plays a key role in reducing porosity near the fusion line. The reason is that an appropriate amount of La_2_O_3_ can promote gas discharge by improving the convection of the molten pool. The molten pool with less rare earth oxide content has poor fluidity, so the gas molecules inside the alloy layer cannot be discharged within a very short cooling time. In paper [16], a similar experimental phenomenon was observed when the Ni60 alloy was fabricated by laser cladding on the aluminum alloy surface. That is, with the addition of rare earth oxides, the number of pores on the side of the alloy layer close to the substrate is significantly reduced. The mechanism is that the rare earth oxide improves the convection of the molten pool and promotes the gas discharge during the melting process.

**Figure 4:**
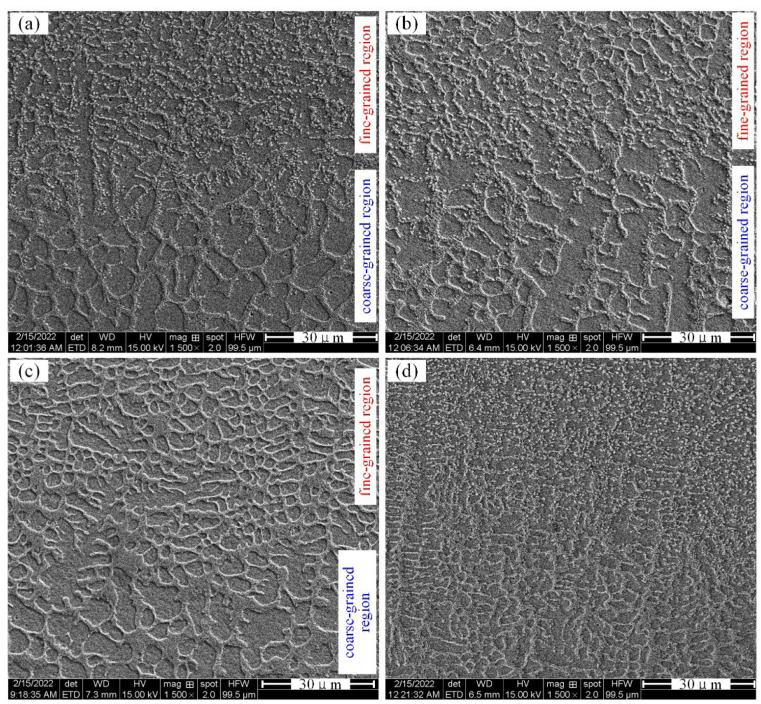
The internal microstructure of AM alloy. (**a**) Specimen-1, (**b**) Specimen-2, (**c**) Specimen-3, (**d**) Specimen-4.

**Figure 5:**
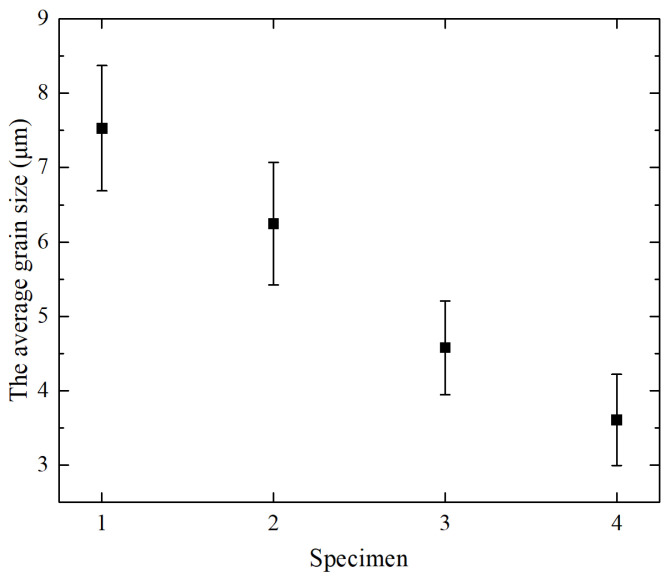
The average grain size of AM alloy.

The internal microstructure of AM alloy is divided into a coarse-grained region at the interface between the layers and a fine-grained region in the middle of each layer, as shown in Figure 4. It is mainly composed of dendrites, cellular dendrites and eutectic. There is the influence of melting and remelting at the bonding interface between AM alloy layers. The grains are remelted and connected to grow, so the grains and the structure become coarse. Furthermore, the addition of La_2_O_3_ significantly affects the microstructural characteristics of AM alloys. Whether it is in the coarse-grained region or the fine-grained region, the grains of AM alloy are significantly refined after adding rare earth oxide. That is, as the mass fraction of La_2_O_3_ increases, the dendrite size decreases significantly, the number of equiaxed crystals increases and the dendrite gap decreases. As shown in Figure 4d, when the rare earth oxide content is 2.0 wt%, the microstructure of the specimen becomes more uniform. The boundary between the coarse-grained region and the fine-grained region becomes indistinct. The average grain size change after adding lanthanum oxide is shown in Figure 5. It can be clearly seen that, with the increase of the mass fraction of lanthanum oxide, the grain size gradually decreases. The grain size of Specimen-4 is about half of that of Specimen-1.

### 3.2. EDS Analysis

**Figure 6:**
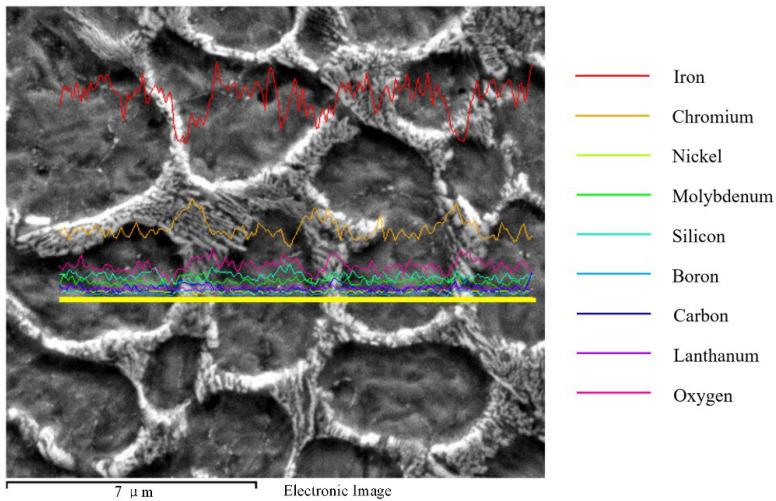
AM alloy line scan EDS curve (Specimen-4).

The intergranular energy spectrum analysis of AM alloy was carried out using EDS line scanning, as shown in Figure 6. The curve representing the iron content decreases significantly at the position of the primary dendrite. The curve representing the chromium element is significantly elevated at the position of the interdendritic eutectic. Due to the absence of gas shielding measures in the AM process, a certain degree of oxidation occurs during the melting of the AM alloy. Therefore, the presence of a small amount of oxygen element is observed in the energy spectrum curve, and the oxygen content in the interdendritic eutectic is higher.

**Figure 7:**
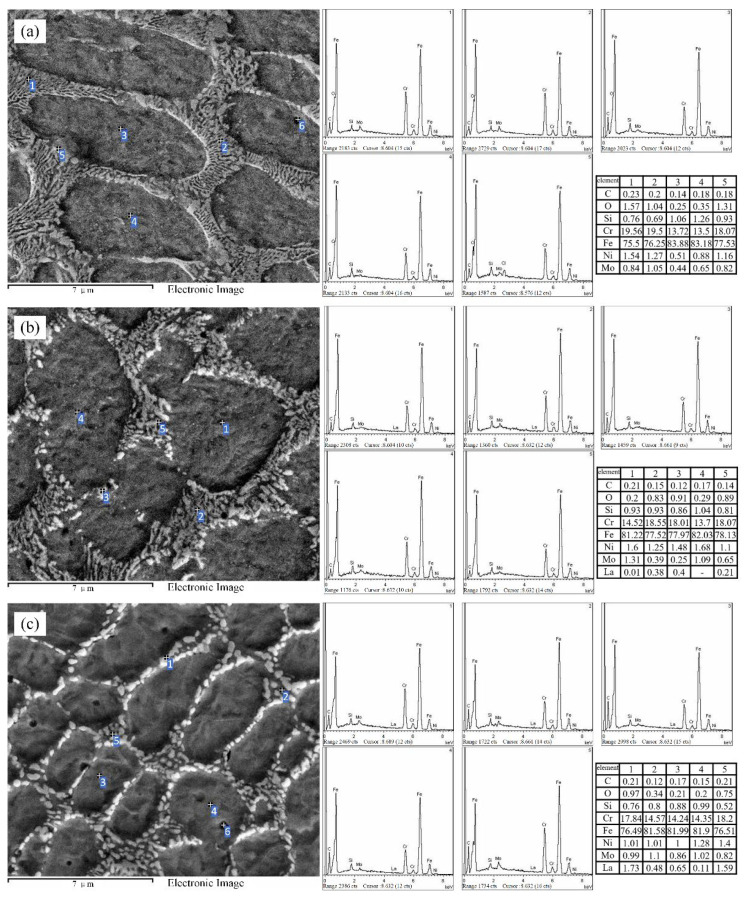
AM alloy point scan EDS curve (**a**) Specimen-0, (**b**) Specimen-1, (**c**) Specimen-4. EDS point scanning.

In order to further analyze the element and phase distribution, quantitative analysis of the AM alloy energy spectrum was carried out using EDS point scanning. Figure 7a shows the element distribution of Specimen-1. As can be seen from the figure, compared with test points 3 and 4, the content of Cr element at points 1, 2 and 5 is significantly increased, and the mass fraction is higher than 18%, while the content of Fe element is reduced. The energy spectrum analysis of Specimen-2 can detect the weak La element, as shown in Figure 7b. The distribution rules of other elements are similar to those in Figure 7a. Figure 7c is the point scan energy spectrum of Specimen-4. From the element content data at points 1 and 5, it can be seen that the La element is concentrated in the interdendritic eutectic, and the Cr element content is also high here. Therefore, based on the element distribution characteristics observed by the point scan and line scan energy spectrum, combined with the X-ray diffraction pattern in the literature [28,29,30], it can be inferred that the main phases composing the primary dendrites are martensite, Fe-Cr and Fe9.7Mo0.3. The phases of the interdendritic eutectic are mainly Cr9.1Si0.9, Fe0.64Ni0.36 and compounds containing La.

From Figure 3, Figure 4 and Figure 5, it can be seen that, by adding La_2_O_3_, the grains of AM alloys are significantly refined. The main cause of grain refinement is the addition of La_2_O_3_. The La element with a larger atomic radius (R_La_ = 0.1877 nm) is a surface-active element. It is easy to react with other elements during the laser additive process, such as Cr, C, Si, Ni, etc. Some stable compounds can be generated, such as Cr3NiB, CeFeSi, LaCrO4, LaNi8C2, etc. [31]. During the liquid phase solidification process, some compounds will float on the surface of the liquid phase and solidify before the liquid metal, which plays the role of slag formation. Therefore, AM alloy is purified by deoxidation and desulfurization, and the inclusion content is reduced. On the other hand, some of the formed compounds and La_2_O_3_ themselves can be used as nuclei for heterogeneous nucleation. The increase in the number of nuclei in the alloy molten pool during the nucleation process helps to promote the nucleation rate and enhance non-spontaneous nucleation [32]. The more nuclei, the finer the fusion grains. In addition, it can be seen from Figure 6 and Figure 7 that the lanthanum element is mainly distributed at the grain boundaries. The La atoms and other compounds located at the grain boundaries will drag the grain boundaries during the grain growth process. Therefore, grain growth is suppressed and the grains are further refined.

### 3.3. Hardness

**Figure 8:**
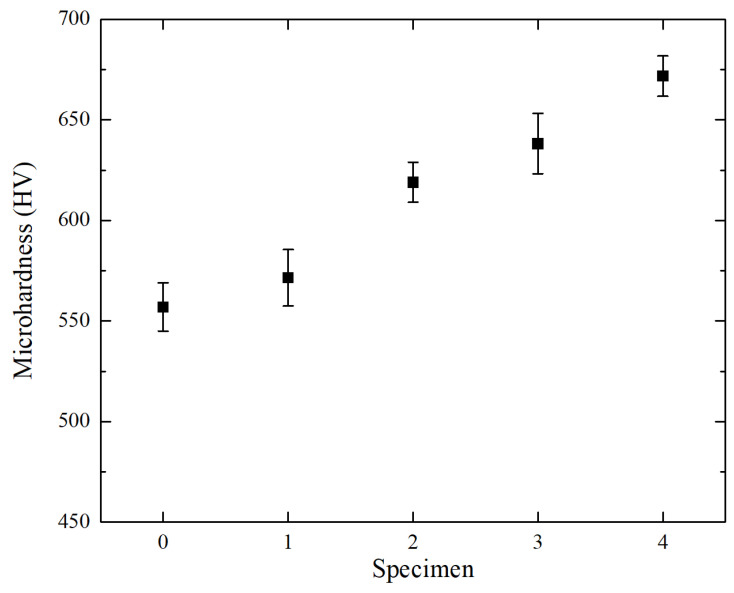
Microhardness of AM alloys.

Figure 8 illustrates the microhardness of AM alloys with different La_2_O_3_ mass fractions. As shown in Figure 8, the average microhardness of Specimens 1 to 4 is 557 ± 12 HV, 571 ± 14 HV, 619 ± 10 HV, 638 ± 15 HV, 671 ± 10 HV, respectively. This indicates that the microhardness of the AM alloy increases gradually after adding La_2_O_3_. When the mass fraction of La_2_O_3_ is 2%, the microhardness of the alloy is the highest. Compared to the alloy without lanthanum oxide, the hardness is increased by about 20%. Generally speaking, the strength and hardness of an alloy increase as the grain size decreases. As shown in Figure 5, the grain size of the AM alloy was significantly reduced after adding La_2_O_3_. This is the reason why the hardness gradually increases as the content of La_2_O_3_ increases.

### 3.4. Friction and Wear Properties Characterization

#### 3.4.1. Coefficient of Friction

**Figure 9:**
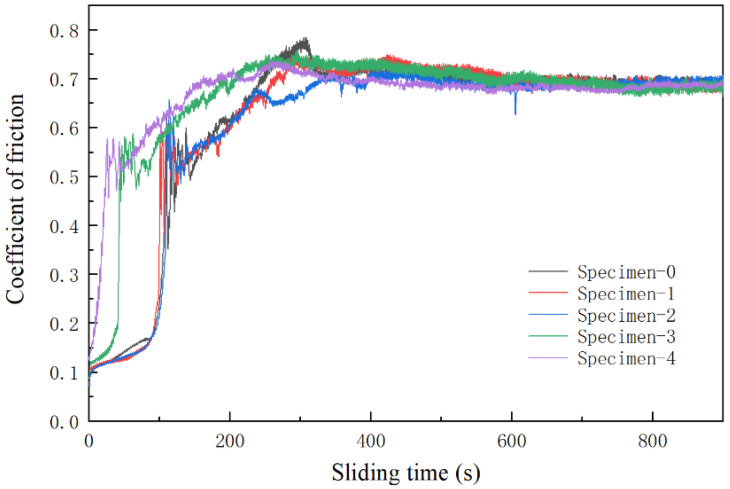
The coefficient of friction of AM alloys.

The coefficient of friction curve of each AM sample automatically recorded by the friction tester is shown in Figure 9. It can be observed from the coefficient of friction curve that the friction test process of AM alloy samples is divided into two parts: the running-in period and the stable friction period. During the running-in period, due to the large surface roughness value of the dual surface, the actual contact area is small, the number of contact points is small, the area of most contact points is large and the contact points are seriously adhered, so the friction coefficient fluctuates greatly and the wear rate is large. With the progress of the friction process, the peaks of the surface micro-peaks are gradually removed, the actual contact area increases, the number of contact points increases and the friction coefficient becomes stable, that is, the stable friction period is entered. In general, the curve of friction coefficient versus time can intuitively reflect the stability of the wear process. The friction coefficient fluctuates smoothly over time, indicating that the friction process is stable. Otherwise, the friction process is unstable. It can be seen that Specimen-0 has a severe running-in period. The coefficient of friction increases significantly with time. The maximum value is reached around 320 s. Then, it begins to descend and enters the stable friction stage. The friction coefficient of Specimen-0 is 0.73. The Specimen-1 also undergoes a relatively obvious running-in stage at the initial stage of friction, but the upward trend of the friction coefficient slowed down, and the peak value of the friction coefficient in the running-in stage is significantly lower than that of Specimen-0. The friction coefficient of Specimen-1 is 0.71. The running-in period of Specimen-2 is more gentle. There is a slight decrease in the coefficient of friction, which is 0.70. With the further increase of La_2_O_3_ content, the running-in period is obviously shortened. The friction coefficient of Specimen-3 begins to increase sharply around 42 s, rises slowly after the running-in period and then enters the stable friction stage. The coefficient of friction of Specimen-4 rises rapidly at the beginning of the relative slip and fluctuates greatly after 28 s, then slowly rises. After 200 s, it enters the stable friction stage. Its friction coefficient is 0.68. In general, the smaller the coefficient of friction, the better the wear reduction performance. Therefore, the wear reduction performance of AM alloys increases with increasing La_2_O_3_ content. In short, adding lanthanum oxide significantly shortens the running-in period of the alloy samples and makes the samples quickly enter the stable friction stage. This is a significant improvement in the friction properties of the alloy after adding lanthanum oxide. With the increase of lanthanum oxide content, the friction coefficient tends to decrease, but the decrease is not obvious.

#### 3.4.2. Wear Scar Profile and Wear Rate

**Figure 10:**
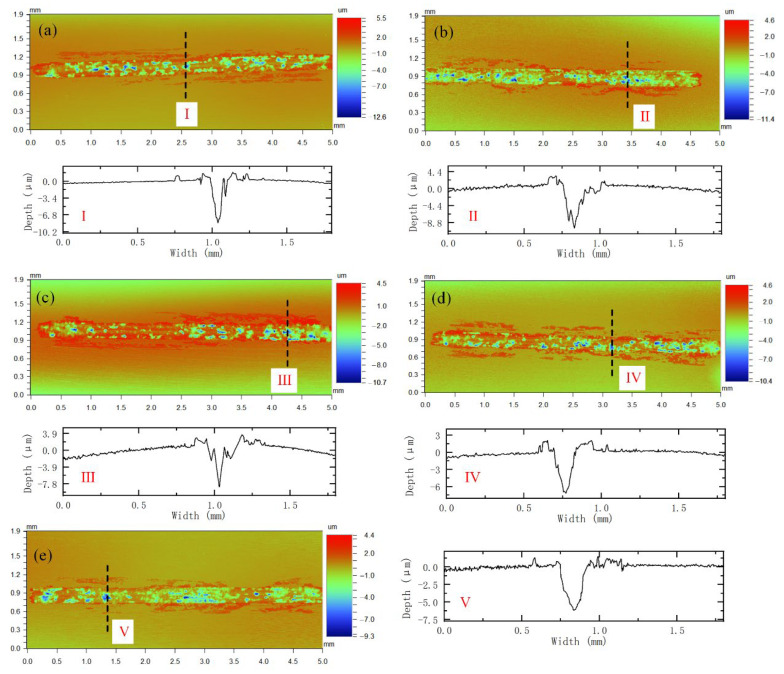
Surface topography and the cross section of the wear tracks (**a**) Specimen-0, (**b**) Specimen-1, (**c**) Specimen-2, (**d**) Specimen-3, (**e**) Specimen-4.

Figure 10 shows the surface topography and representative cross-section of the wear scar. The (I)–(V) in the figure are the cross-sectional profiles of the relatively deep positions in each wear scar, and the positions are shown by the dash lines. It can be seen that the deepest wear scar of Specimen-0 is 12.6 μm, and that of Specimen-1 is reduced to 11.4 μm. With the addition of La_2_O_3_, the wear scar depth decreases gradually. The maximum depth of Specimen-4 is reduced to 9.3 μm.

In addition, the average width, depth and wear rate of the wear scars (characterized by volume loss) were measured by the profile profiles, as shown in Table 2. According to the measured data, the width of the wear scar decreases as the mass friction of La_2_O_3_ increases. The maximum width of the wear scar is 452 mm (Specimen-0), and the minimum width is 372 mm (Specimen-4). The variation trend of wear scar depth is the same as that of wear scar width. As the mass fraction of La_2_O_3_ increases, the average depth of the wear scar decreases significantly. The wear scar depth of Specimen-0 reached 7.24 μm, and that of Specimen-4 decreased to 4.99 μm. The change in the wear rate also showed the same trend, that is, the wear rate decreases significantly as the mass fraction of La_2_O_3_ increases. Compared with Specimen-0, the wear rate of Specimen-4 is reduced by 38%. Generally speaking, the smaller the wear rate under the same conditions, the better the wear resistance. Therefore, the wear resistance of AM alloy is improved after adding La_2_O_3_, and, as the mass fraction of La_2_O_3_ increases, the wear resistance improves. It can be seen that the purpose of preparing iron-chromium alloy powder for laser additive manufacturing with high wear resistance is achieved by adding rare earth oxides. The reasons for the improved wear resistance of the alloy and the wear mechanism will be explained in the following paragraphs.

#### 3.4.3. Wear Mechanism

**Figure 11:**
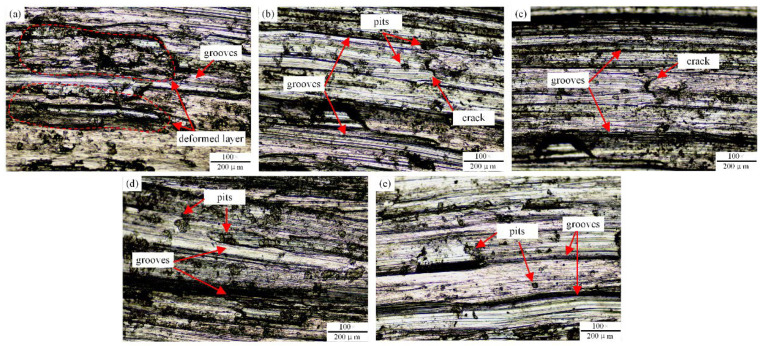
Microscopic morphology of the wear track of AM alloy. (**a**) Specimen-0, (**b**) Specimen-1, (**c**) Specimen-2, (**d**) Specimen-3, (**e**) Specimen-4.

In order to reveal the wear mechanism, the wear morphology characteristics of the AM alloy specimens after friction and wear tests were observed by OM, as shown in Figure 11. Since the surface hardness of AM alloy without La_2_O_3_ (Specimen-0) is much lower than that of the counter-grinding material, the surface material is sheared and transferred by the counter-grinding material during the wear process. Obvious plastic deformation, transfer film and a small amount of grooves morphology can be seen on the surface of the test area, which is shown in Figure 11a. Therefore, the main wear forms of Specimen-0 under the self-lubricating wear condition at room temperature are adhesive wear and abrasive wear.

After adding La_2_O_3_, AM alloy specimens produce the morphology shown in Figure 11b–e along the wear direction. The surfaces of each sample have grooves and scratches that are parallel to each other. This is because the abrasive grains or asperities on the surface of the counter-grinding pair are easily embedded in the specimen surface layer and form grooves and scratches with the movement of the counter-grinding pair. At the same time, some scattered wear debris particles and surface oxide particles are observed on the wear surface. No significant plastic deformation area was observed. Therefore, the wear form of AM alloy after adding La_2_O_3_ is mainly abrasive wear. As the mass fraction of La_2_O_3_ increases, the depth and width of the groove show a decreasing trend. In addition, pits and microcracks formed after the bulk exfoliation can be observed. Under the fatigue effect of the reciprocating cyclic tangential load and the cutting effect, the surface material is affected by the shear stress on the grinding pair, which leads to the peeling of the binder phase and then causes the block peeling. Therefore, the wear pattern of AM alloy after adding La_2_O_3_ also manifests as fatigue spalling of hard particles. The worn surface topography of each sample shown in Figure 11 verifies that, as the mass friction of La_2_O_3_ increases, the wear resistance and wear reduction of AM alloy show an increasing trend.

Generally, fine-grained metals have higher strength and hardness than coarse-grained metals. Therefore, the grain refinement after adding La_2_O_3_ is the leading cause of the improvement of the wear resistance of AM alloy—this is the fine-grain strengthening effect. When the alloy is subjected to external force, the plastic deformation of the microstructure with finer grains can be dispersed into more grains. As a result, the plastic deformation is more uniform and produces fewer stress concentrations. Therefore, compared with coarse-grained metals, fine-grained metals have a higher ability to locally resist the indentation of hard objects into their surfaces under the same external force. In addition, more grain boundaries after grain refinement make it more difficult for dislocations, especially misaligned dislocations, to transfer to other grains, and disordered grain boundaries make slip more difficult. The finer the microstructure of the alloy—that is, the smaller the grain size—the more the grain boundary will hinder the movement of dislocations, and the more difficult it will be for crack propagation. Grain refinement generally also increases material toughness. Therefore, the increase in strength and toughness caused by grain refinement increases the wear resistance of AM alloy. In paper [33], laser cladding layers of nickel-chromium-iron alloys with rare earth oxide were prepared. The friction test was carried out under the condition of oil lubrication. Although the friction conditions are different from those in this study, the main reason for the improved wear resistance is still attributed to the refined grain size and the increased density of solid solubility after the addition of rare earth oxides.

## 4. Conclusions

The effect of adding La_2_O_3_ on the microstructure, friction and wear performance and wear mechanism of AM iron-chromium alloy was systematically investigated. The following results were obtained.

(1) By adding La_2_O_3_, the microstructure of AM alloy becomes more uniform and the grains are significantly refined. La element is the main cause of grain refinement. La element easily reacts with other elements to form some stable compounds during the laser additive process. It can perform slag formation and reduce the content of alloy inclusions. In addition, some of the compounds formed and the La_2_O_3_ themselves can be used as nuclei for heterogeneous nucleation. Grain refinement is further promoted.
(2) The running-in period is significantly shortened after adding La_2_O_3_. The coefficient of friction is reduced. The wear rate has dropped significantly. This shows that the wear reduction and wear resistance of AM alloys have been improved. The increase in the strength and toughness of the alloy caused by grain refinement after the addition of La_2_O_3_ is the main reason for the improvement of the wear resistance of the AM alloy.
(3) The wear form of AM alloys after adding rare earth oxide becomes abrasive wear, accompanied by the spalling of hard particles. This is mainly due to the grain refinement strengthening effect after adding rare earth oxide.

The scope of this research is limited to the influence of lanthanum oxide addition on the microstructure and wear performance of AM alloys. The effect of adding La_2_O_3_ on mechanical properties, such as tensile and bending, would be an interesting future research subject.

## Figures and Tables

**Table 1:** Chemical composition of iron-chromium alloy powder (wt%).

Element	C	Si	Mo	Cr	Ni	B	Fe
Compositions	0.15	1.1	1.0	17.35	1.35	≤1.26	balance

**Table 2:** Wear properties of AM alloy.

Scheme	Width (μm)	Depth (μm)	Wear Rate (10^−4^ mm^3^/Nm)
0	452 ± 20.3	7.24 ± 0.32	3.599 ± 0.56
1	413 ± 28.2	6.65 ± 0.30	3.332 ± 0.41
2	399 ± 26.4	6.07 ± 0.59	3.051 ± 0.42
3	375 ± 30.2	5.40 ± 0.43	2.453 ± 0.31
4	372 ± 21.5	4.99 ± 0.38	2.217 ± 0.35

## Data Availability

The data presented in this study are available on request from the corresponding author.
